# Rosmarinic Acid-Rich *Perilla frutescens* Extract-Derived Silver Nanoparticles: A Green Synthesis Approach for Multifunctional Biomedical Applications including Antibacterial, Antioxidant, and Anticancer Activities

**DOI:** 10.3390/molecules29061250

**Published:** 2024-03-12

**Authors:** Vasudeva Reddy Netala, Tianyu Hou, Siva Sankar Sana, Huizhen Li, Zhijun Zhang

**Affiliations:** School of Chemistry and Chemical Engineering, North University of China, Taiyuan 030051, Chinahzli@nuc.edu.cn (H.L.)

**Keywords:** *Perilla frutescens*, rosmarinic acid, AgNPs, DLS, cytotoxicity, A549

## Abstract

This study describes a simple, cost-effective, and eco-friendly method for synthesizing silver nanoparticles using a rosmarinic acid extract from *Perilla frutescens* (PFRAE) as the bioreduction agent. The resulting nanoparticles, called PFRAE-AgNPs, were characterized using various analytical techniques. The UV–Vis spectrum confirmed the formation of PFRAE-AgNPs, and the FTIR spectrum indicated the participation of rosmarinic acid in their synthesis and stabilization. The XRD pattern revealed the crystal structure of PFRAE-AgNPs, and the TEM analysis showed their spherical morphology with sizes ranging between 20 and 80 nm. The DLS analysis indicated that PFRAE-AgNPs were monodispersed with an average diameter of 44.0 ± 3.2 nm, and the high negative zeta potential (−19.65 mV) indicated their high stability. In the antibacterial assays, the PFRAE-AgNPs showed potent activity against both Gram-positive (*Bacillus subtilis* and *Staphylococcus aureus*) and Gram-negative (*Escherichia coli* and *Pseudomonas aeruginosa*) bacterial pathogens, suggesting that they could be used as a potential antibacterial agent in the clinical setting. Moreover, the antioxidant activity of PFRAE-AgNPs against DPPH and ABTS radical scavengers highlights their potential in the treatment of various oxidative stress-related diseases. PFRAE-AgNPs also demonstrated significant anticancer activity against a range of cell lines including human colon cancer (COLO205), human prostate carcinoma (PC-3), human lung adenocarcinoma (A549), and human ovarian cancer (SKOV3) cell lines suggesting their potential in cancer therapy. The nanoparticles may also have potential in drug delivery, as their small size and high stability could enable them to cross biological barriers and deliver drugs to specific target sites. In addition to the aforementioned properties, PFRAE-AgNPs were found to be biocompatible towards normal (CHO) cells, which is a crucial characteristic for their application in cancer therapy and drug delivery systems. Their antibacterial, antioxidant, and anticancer properties make them promising candidates for the development of new therapeutic agents. Furthermore, their small size, high stability, and biocompatibility could enable them to be used in drug delivery systems to enhance drug efficacy and reduce side effects.

## 1. Introduction

**Figure 1:**
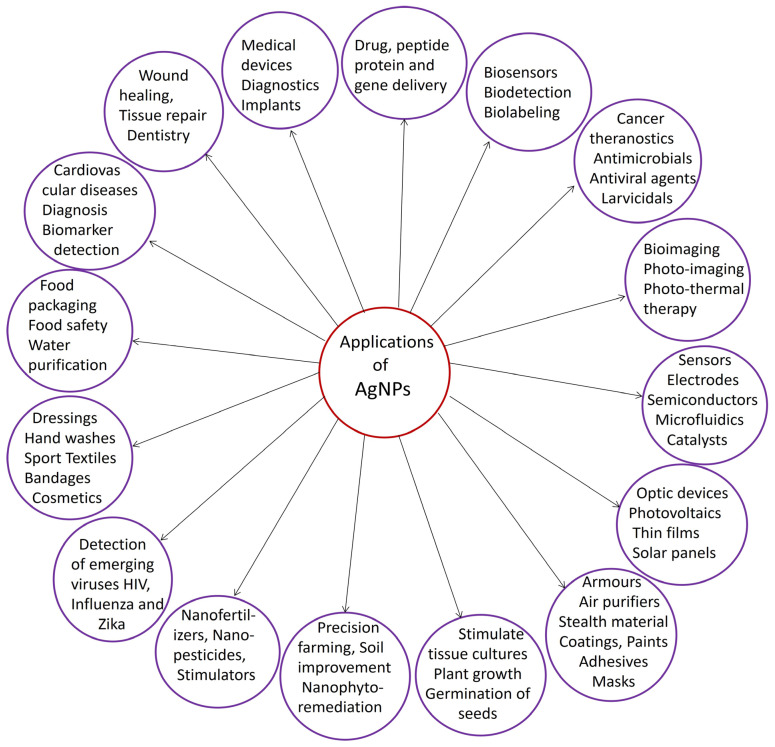
A diagram depicting diverse applications of silver nanoparticles.

Nanoparticles, which range from 1 to 100 nm, have received a great deal of attention due to their unique physicochemical and optical properties [1]. Currently, extensive research is being conducted on silver nanoparticles (AgNPs) due to their wide range of potential applications. AgNPs can be used in various materials, including optical sensors [2], electrical batteries [3], supercapacitors [4], nanoceramics [5], flame retardants [6], biosensors [7], drug delivery vehicles [8], catalysts [9], and as antimicrobial, antioxidant, anti-inflammatory, and anticancer agents [10,11,12]. Due to their antibacterial properties, AgNPs have found widespread use in various applications, including dental implants, surgical devices, bone and tissue engineering applications, and other biomedical devices [13,14,15]. AgNPs have also been incorporated into food packaging, paints, and coatings [16,17]. AgNPs have been shown to protect against a range of oxidative stress-related diseases, including Alzheimer’s, Parkinson’s, and cardiovascular disease [18,19,20]. AgNPs have shown promise as potential anticancer agents due to their ability to induce apoptosis (cell death) in cancer cells. Additionally, AgNPs have been shown to have low toxicity towards normal cells, making them a potential alternative to conventional chemotherapy drugs [21,22,23]. AgNPs have been investigated as potential drug delivery vehicles due to their small size and unique physicochemical properties. They can be easily functionalized with different molecules, such as drugs or targeting ligands, to improve their specificity and efficacy [23,24,25]. Overall, AgNPs have shown great potential in various applications (Figure 1), and continued research in this area is needed to fully understand their mechanisms of action and optimize their use in different fields. 

**Figure 2:**
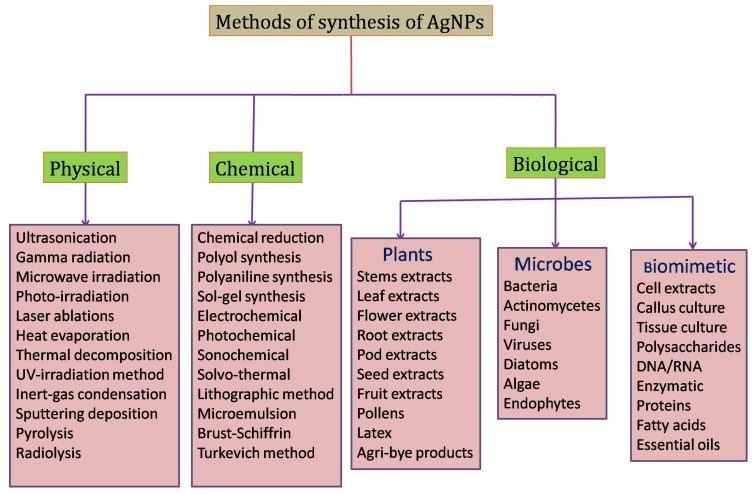
Illustration depicting various methods for the synthesis of silver nanoparticles.

Various physical and chemical methods, such as the evaporation–condensation method [26], solvothermal synthesis [27], chemical reduction method [28], irradiation methods including gamma irradiation [29], ultrasound method [30], UV-irradiation [31], and microwave methods [32], lithography [33], electrochemical method [34], Tollen’s method [35], polyaniline [36], thermal decomposition [37], and laser ablation methods [38], have been reported for the synthesis of AgNPs. While these approaches are highly productive, they involve tedious and time-consuming processes and are not eco-friendly [39,40]. Different methods for the synthesis of AgNPs are clearly represented (Figure 2). 

Green synthesis, a process that utilizes natural resources and environmentally friendly methods, has gained significant attention in the fabrication of metal and metal oxide nanoparticles, including Zn, ZnO, Cu, CuO, Ti, TiO_2_, Mg, and MgO [41]. Drawing inspiration from nature’s own mechanisms, green synthesis harnesses the power of various natural extracts, such as those from plants, algae, fungi, bacteria, as well as enzymatic reactions, polysaccharides, essential oils, natural honey, and pollens [42,43,44]. These green synthesis approaches offer a sustainable and eco-friendly alternative to conventional chemical methods. In green synthesis, the inherent reducing and stabilizing properties of phytochemicals, enzymes, polysaccharides, and other natural compounds present in these extracts play a crucial role in the fabrication of nanoparticles. These bioactive components serve as both bioreductants and stabilizers, facilitating the precise and eco-friendly synthesis of metal and metal oxide nanoparticles [45,46]

Among the numerous applications of green synthesis, its role in the production of AgNPs stands out prominently, particularly in the biomedical and environmental sectors. Green synthesis replicates the way living organisms create and utilize nanoparticles, resulting in nanoparticles with remarkable properties ideal for various applications [42,43,44,45,46]. In biomedical contexts, green synthesized AgNPs hold immense promise due to their unique attributes aligned with the stringent requirements of biocompatibility, safety, and efficacy in medical applications. These nanoparticles are eco-friendly, exhibiting low toxicity and excellent stability, making them suitable candidates for drug delivery, wound healing, diagnostic imaging, and antimicrobial therapies [47,48,49].

The use of green synthesis approaches, such as those utilizing plant extracts, algae, fungi, bacteria, enzymatic reactions, polysaccharides, essential oils, natural honey, and pollens, aligns with the principles of green chemistry and sustainability [48,49]. Unlike conventional chemical methods that involve hazardous and toxic chemicals, green synthesis eliminates the need for such harmful substances, reducing environmental and health risks during production and disposal [50,51]. Furthermore, green synthesis promotes sustainability by repurposing natural resources, reducing waste, and minimizing the carbon footprint of the synthesis process. The scalability and cost-effectiveness of green synthesis make it an economically viable option for both research and industrial applications. By efficiently utilizing natural resources and promoting sustainable practices, green synthesis contributes to the advancement of eco-friendly nanotechnology [47,48,49,50,51].

Plant extracts contain various phytochemicals, such as polyphenols, flavonoids, terpenoids, and alkaloids, some of which have strong reducing properties. For instance, flavonoids and phenolic compounds present in plants can donate electrons to silver ions (Ag+), leading to the reduction of Ag+ to AgNPs [52]. This reduction process is often initiated by the interaction between the active compounds and silver ions, leading to the nucleation and growth of nanoparticles [53]. Using plant extracts as bioreductants eliminates the need for harsh chemical reductants that are typically used in conventional methods. This reduces the risk of generating harmful byproducts and minimizes environmental pollution [54]. Plant extracts also serve as effective stabilizing agents for AgNPs. Phytochemicals present in the extracts, such as polyphenols, can adsorb onto the nanoparticle surface through electrostatic interactions and hydrogen bonding or Van der Waals forces. This adsorption helps prevent the agglomeration or aggregation of AgNPs, ensuring their stability over time [55,56]. This stability is crucial for ensuring the nanoparticles’ uniform distribution and long-term effectiveness in various applications, including biomedical and catalytic ones [55,56,57]. Stabilizing agents derived from plant extracts help maintain the small particle size and prevent precipitation, ensuring the nanoparticles remain dispersed [57,58]. The absence of toxic residues enhances the biocompatibility of AgNPs synthesized with plant extracts [59,60]. This is particularly advantageous in biomedical applications, such as drug delivery and imaging, where the safety of nanoparticles is paramount [61]. The biocompatibility of AgNPs synthesized using plant extracts makes them well-suited for medical and biological applications. These nanoparticles can be used in drug delivery systems, wound healing, tissue engineering, and as antimicrobial agents without adverse effects on living organisms [62,63]. 

*Perilla frutescens*, belonging to the family Lamiaceae, is widely distributed in East Asian countries such as Japan, China, Korea, and Vietnam [64]. *P. frutescens*, an important medicinal plant possesses different biological activities including anti-inflammatory, antioxidant, antidepressant, antiallergic, antimicrobial, antidiabetic, insecticidal, cardioprotective, neuroprotective, and hepatoprotective effects [65,66,67,68]. The different biological activities are due to the presence of active secondary metabolites in the leaves of *P. frutescens*. The secondary metabolites include flavonoids, polyphenolic compounds, terpenoids, quinones, alkaloids, coumarins, anthocyanins, carotenoids, etc. [65]. The leaves of *P. frutescens* are rich in polyphenolic compounds among all the other secondary metabolites. These include rosmarinic acid, caffeic acid, protocatechuic acid, chlorogenic acid, vanillic acid, isovanillic acid, sinapic acid, gallic acid, 4-coumaric acid, sagerinic acid, ferulic acid, and cimidahurinine [65,66,67,68]. Rosmarinic acid (RA) is one of the major secondary metabolites present in the leaves of *P. frutescens* [69]. RA, a polyphenolic compound, possesses strong anti-inflammatory properties. RA reduces inflammatory conditions such as allergic rhinitis, atopic dermatitis, arthritis, asthma, etc. [70,71,72]. RA shows hepatoprotective effects and is used as pharmaceutical ingredient in the treatment of liver diseases including hepatocellular carcinoma, hepatitis, liver cirrhosis fibrosis, etc. [73]. RA shows neuroprotective effects and prevents neurodegeneration by inhibiting nitric oxide protection [74]. RA exerts antidepressant effects via upregulation of brain-derived neurotrophic factor, downregulation of mitogen-activated protein kinase phosphatase-1 (Mkp-1), suppression of corticosterone synthesis, and increased dopamine synthesis [75]. RA shows cardioprotective effects against acute myocardial infarction and arrhythmia by the suppression of the NF-kB inflammatory signaling pathway and ROS production [76]. RA exhibits anticancer effects against different cancer cells including colon cancer, melanoma, breast cancer, etc. [77]. 

In this study, we utilized an ultrasound-assisted extraction method to prepare a rosmarinic acid extract from the leaves of *P. frutescens*. The extracted material was then purified and analyzed using HPLC, and subsequently named *P. frutescens* rosmarinic acid extract (PFRAE). PFRAE was then used as a bioreduction agent for the synthesis of AgNPs. The resulting PFRAE-AgNPs were characterized using a variety of biophysical techniques, including ultraviolet–visible (UV–Vis) spectroscopy, Fourier transform infrared (FTIR) spectroscopy, X-ray diffraction (XRD), transmission electron microscopy (TEM), and dynamic light scattering (DLS). Antibacterial activity of PFRAE-AgNPs was evaluated against both Gram-positive (*Bacillus subtilis* and *Staphylococcus aureus*) and Gram-negative (*Escherichia coli* and *Pseudomonas aeruginosa*) bacterial species. Antioxidant activity of PFRAE-AgNPs was assessed using 1,1-diphenyl-2-picrylhydrazyl (DPPH) and 2,2′-azino-bis(3-ethylbenzothiazoline-6-sulfonicacid (ABTS) radical scavenging assays. The cytotoxicity of PFRAE-AgNPs was assessed against various cancer cell lines, including human colon cancer (COLO205), human prostate carcinoma (PC-3), human lung adenocarcinoma (A549), human ovarian cancer (SKOV3), as well as a normal cell line (CHO). 

## 2. Results and Discussion

**Figure 3:**
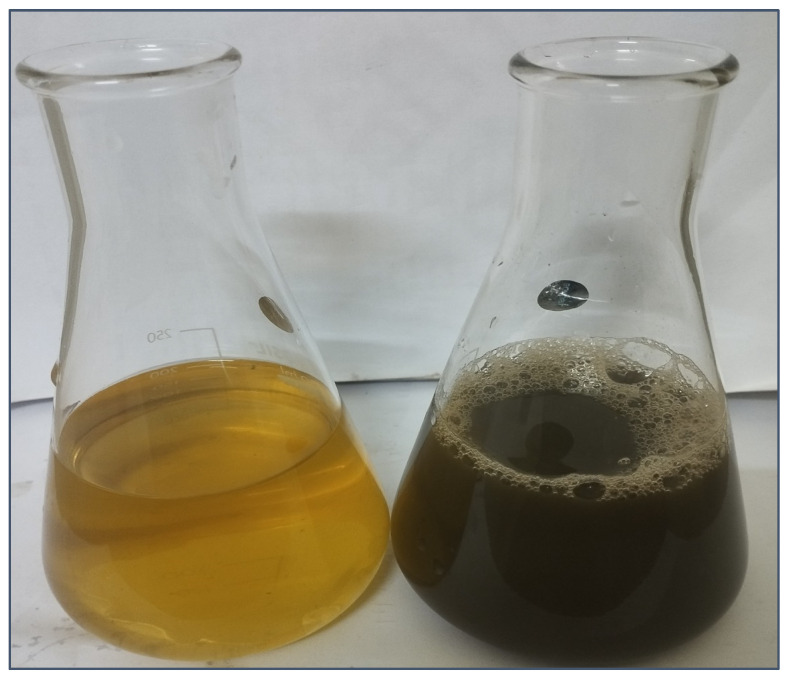
Visualizing the spectacular color transformation: synthesis of PFRAE-AgNPs revealed through a striking transition.

In this study, we employed an ultrasound-assisted extraction method to efficiently extract rosmarinic acid from *P. frutescens* leaves. Subsequently, the resulting extract underwent high-performance liquid chromatography (HPLC) analysis, revealing rosmarinic acid as the predominant compound, with an impressive purity level of 85%. The concentration of rosmarinic acid was found to be 0.798 mg/mL of PFRAE. A total of 10 mL of PFRAE was added to 90 mL of 2 mM AgNO_3_ solution. This mixture was then subjected to boiling for 1 h, followed by a cooling period of 30 min in a dark chamber. During the incubation phase, an intriguing transformation occurred: the initially light-colored reaction solution gradually transitioned to a deep, rich brown hue [55,78] (Figure 3). This observable change in color is a significant indicator of the successful synthesis of nanosilver, which we have aptly named PFRAE-AgNPs. 

The remarkable alteration in color from light to dark brown can be attributed to a phenomenon known as localized surface plasmon resonance (LSPR). LSPR is a distinctive optical property of nanoparticles, such as AgNPs, arising from the collective oscillation of electrons on their surfaces when they interact with light [79,80]. In the case of PFRAE-AgNPs, this shift in color is a strong indication of the successful formation of silver nanoparticles and underscores the exciting nanoscale transformations taking place in our synthesis process.

### 2.1. UV–Visible Spectroscopic Study of PFRAE-AgNPs

**Figure 4:**
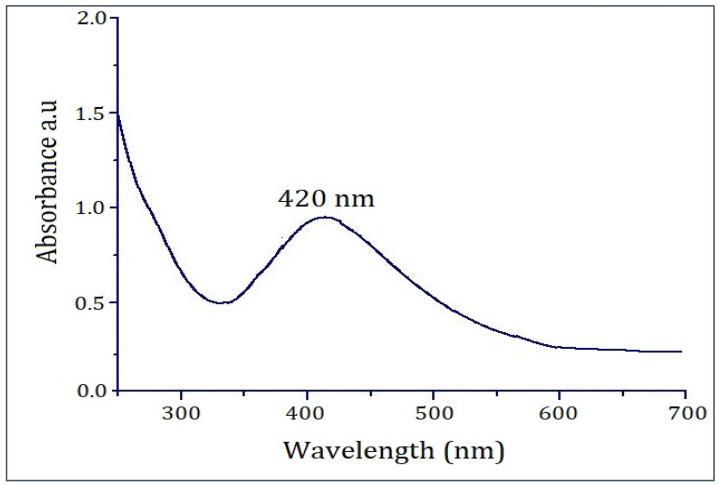
PFRAE-AgNPs exhibit a notable UV–Vis absorption peak at 420 nm, signifying surface plasmon resonance (SPR).

The synthesis was further confirmed by UV–Vis analysis. The UV–Vis spectrum of PFRAE-AgNPs exhibited a distinct absorption peak at 420 nm (Figure 4), which strongly suggests the successful formation of non-aggregated, monodispersed, and small-sized PFRAE-AgNPs. The distinct absorption peak observed at 420 nm in the UV–Vis spectrum of PFRAE-AgNPs is a direct result of the excitation of surface plasmon vibrations induced by the metallic nanoparticles, hence referred to as the surface plasmon resonance (SPR) peak [81]. Notably, this SPR peak serves as a hallmark feature exclusive to AgNPs and underpins their distinctive optical properties, primarily stemming from localized SPR [80,81].

UV–Vis analysis holds a pivotal role in characterizing metallic nanoparticles, particularly AgNPs. It furnishes critical insights into their size, morphology, and concentration [82,83]. A key attribute of the UV–Vis spectrum of AgNPs is the SPR peak, arising from the oscillation of electrons within the nanoparticles’ conduction band in response to the incident light’s electromagnetic field [83,84,85]. The SPR peak’s characteristics are profoundly influenced by the nanoparticles’ size, shape, and composition, thus enabling an in-depth understanding of the physical and chemical attributes of AgNPs [80,81,82].

Furthermore, the SPR peak is acutely sensitive to the immediate nanoparticle environment. This sensitivity renders it a valuable tool for detecting a wide range of analytes, including biological molecules and environmental contaminants [86]. Consequently, UV–Vis analysis of AgNPs, coupled with the discernible SPR peak, assumes critical importance in characterizing and harnessing these materials across diverse domains, such as nanotechnology, biomedicine, and environmental science [87,88].

### 2.2. Functional Group Study of PFRAE-AgNPs Using FTIR Spectroscopy

**Figure 5:**
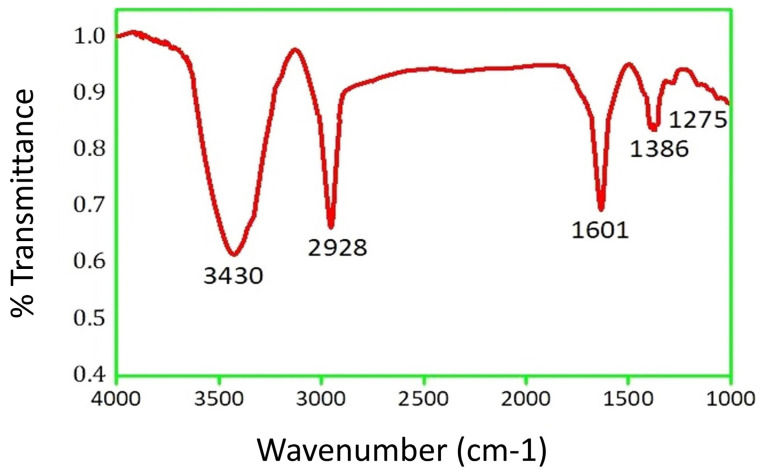
FTIR spectrum of PFRAE-AgNPs, highlighting the presence of diverse functional groups that play a role in the synthesis and stabilization of PFRAE-AgNPs.

Fourier transform infrared spectroscopy (FTIR) is a powerful analytical tool that is used to study the chemical composition and molecular structure of a wide range of materials, including metallic nanoparticles such as AgNPs [89]. FTIR analysis of AgNPs can provide valuable information about the nature of the capping agents or stabilizers that are used during the synthesis of these nanoparticles [90]. These agents play a critical role in stabilizing the nanoparticles and preventing their aggregation, which is essential for their stability and functionality in various applications [91]. FTIR analysis can also reveal the presence of functional groups on the surface of the AgNPs, which can be useful in understanding their interaction with other molecules or materials. In the present investigation, the FTIR spectrum of PFRAE-AgNPs (Figure 5) revealed the functional groups, including 3430, 2928, 1601, 1386, and 1275 cm^−1^. 

The peak at 3430 cm^−1^ is corresponding to O-H stretching vibrations of polyphenolic compounds [92]. The peak at 2928 cm^−1^ could be assigned to C-H stretching of aromatic compounds. The peak at 1601 cm^−1^ is corresponding to stretching vibration of aromatic C=C groups [93]. The peak at 1386 cm^−1^ is corresponding to C=O stretching vibrations of esters. The peak at 1275 cm^−1^ is corresponding to C=O stretching vibrations of aromatic compounds. Rosmarinic acid is a polyphenolic aromatic compound that has carboxylic acid, ester, aromatic alkene, and phenolic functional groups. Hence FTIR clearly demonstrated that PFRAE is involved in the synthesis and stabilization of PFRAE-AgNPs.

### 2.3. XRD Analysis of PFRAE-AgNPs for Crystalline Structure Elucidation

**Figure 6:**
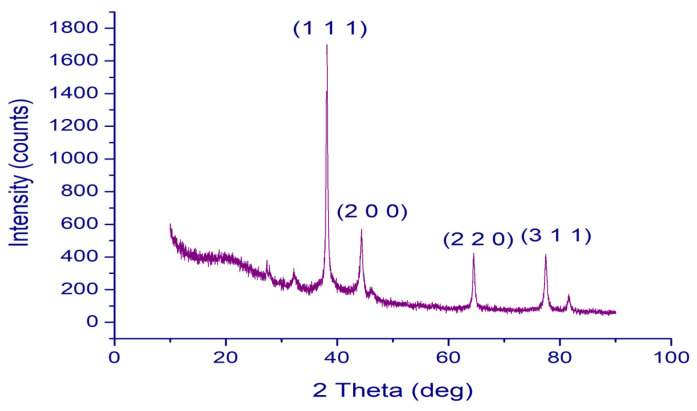
XRD Analysis of PFRAE-AgNPs: crystalline nature confirmed by four distinct peaks (1 1 1), (2 0 0), (2 2 0), and (3 1 1).

The crystal structure of the synthesized PFRAE-AgNPs was determined using X-ray diffraction (XRD) analysis. The XRD pattern of PFRAE-AgNPs revealed four Bragg’s peaks at 38.17, 44.33, 64.51, and 77.45, which corresponded to the planes of (1 1 1), (2 0 0), (2 2 0), and (3 1 1), respectively, indicating a face-centered cubic (FCC) crystal structure (Figure 6).

XRD analysis is an important tool for the characterization of AgNPs, as it provides information on the crystal structure, phase, and orientation of the nanoparticles [94,95]. The analysis can also reveal impurities or secondary phases that may be present in the sample. In the case of PFRAE-AgNPs, XRD analysis provided crucial information on the crystal structure of the nanoparticles, which can affect their physical and chemical properties. The FCC phase of the nanocrystals, as determined by XRD analysis, is an important parameter that can impact their biological activity and interaction with other molecules or materials [96,97]. Overall, XRD analysis is a valuable technique for the characterization and optimization of AgNPs in various applications such as catalysis, sensing, and biomedical devices.

### 2.4. TEM Analysis of PFRAE-AgNPs for Size and Morphology

**Figure 7:**
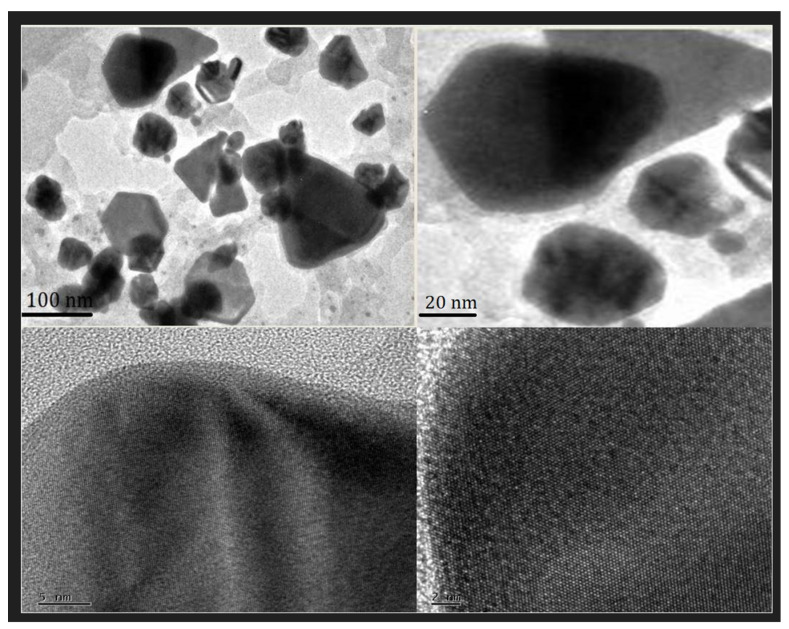
TEM micrograph at a 100 nm scale shows the anisotropic nanoparticles including spherical, rhombic, trigonal, and rectangular shapes. HR-TEM at 5 nm and 2 nm clearly represents crystal lattice fringes and rough surface morphology.

Transmission electron microscopy (TEM) analysis was performed to determine the size and shape of PFRAE-AgNPs. The TEM images revealed that the synthesized nanoparticles were in the size range of 20–80 nm and had different shapes such as spherical, rhombic, trigonal, and rectangular shapes, with most of the nanoparticles being spherical in morphology (Figure 7). TEM analysis is an essential tool for the characterization of nanoparticles, as it provides information on their size, morphology, and crystal structure at the nanoscale level [98]. The size and shape of nanoparticles can affect their physical and chemical properties, which in turn can influence their biological activity and performance in various applications [98,99]. TEM analysis allows researchers to visualize and quantify these properties, and to optimize the synthesis conditions to obtain nanoparticles with desired properties. In the case of PFRAE-AgNPs, TEM analysis provided crucial information on the size and shape of the nanoparticles, which can impact their biological activity and interaction with other molecules or materials. Overall, TEM analysis is a valuable technique for the characterization and optimization of AgNPs in various applications such as drug delivery, biosensing, and imaging [98,99,100].

### 2.5. DLS Analysis of PFRAE-AgNPs to Reveal Particle Size Distribution and Zeta Potential Measurement

**Figure 8:**
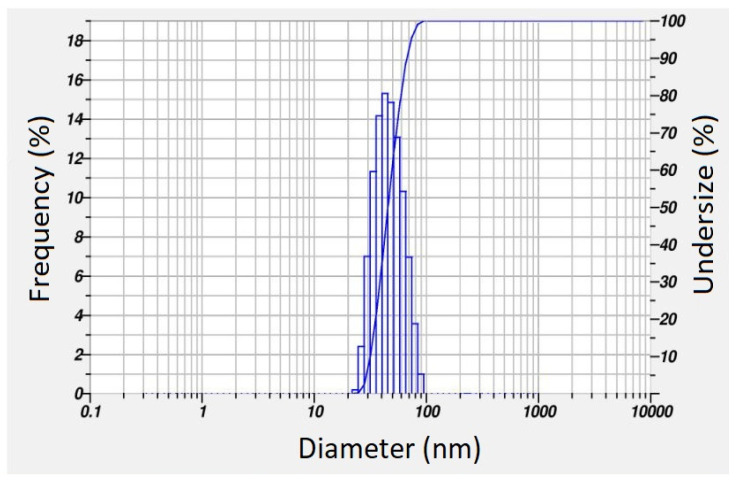
Particle size distribution determined by dynamic light scattering analysis.

Determination of the average hydrodynamic size of PFRAE-AgNPs involves analyzing the time-dependent fluctuations in the intensity of scattered light emitted by the particles through DLS (dynamic light scattering) analysis [101]. This technique capitalizes on the Brownian motion of particles in solution, whereby the speed of their motion and interaction with surrounding solvent molecules inform calculations of their hydrodynamic size [102]. The results of the DLS analysis of PFRAE-AgNPs reveal that particles are distributed within the range of 20–80 nm sizes in water dispersion medium, as illustrated in Figure 8. The average hydrodynamic radius of the PFRAE-AgNPs is 44.0 ± 3.2 nm, indicating that the AgNPs are relatively small. This finding is consistent with their unique physical and chemical properties that make them highly useful in a broad range of applications. Notably, the size distribution of the particles, as evidenced by the standard deviation of 3.2 nm, suggests that there may be some variation in the size of the particles within the sample. This variability could have implications for the effectiveness of the PFRAE-AgNPs in certain applications, and thus, further investigations may be necessary to determine the impact of this size variation on their performance. Overall, the DLS analysis provides valuable insights into the hydrodynamic properties of the PFRAE-AgNPs, which have promising potential for various scientific and technological applications [103].

Upon analyzing the polydispersity index (PDI) value, it was determined that the PFRAE-AgNPs were monodispersed, with a PDI value of 0.413. This suggests that the nanoparticle size distribution was relatively narrow and uniform, indicating the quality of the synthesis process. The monodispersity of PFRAE-AgNPs, as indicated by the low PDI value of 0.413, is of great significance in pharmaceutical applications. In drug delivery, the uniform size and shape of nanoparticles can affect their circulation time in the bloodstream, cellular uptake, and overall therapeutic efficacy [101,102,103]. The use of monodispersed nanoparticles can improve the reproducibility and reliability of drug delivery systems, making them more effective in treating diseases. Therefore, the PDI value serves as an important parameter in assessing the quality and suitability of AgNPs for various pharmaceutical applications [101,102,103,104]. 

**Figure 9:**
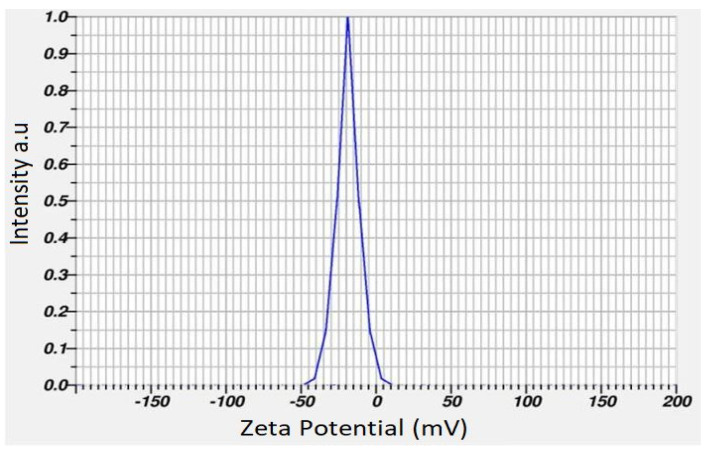
Zeta potential measurement using the dynamic light scattering technique.

DLS analysis revealed that the zeta potential value of PFRAE-AgNPs was found to be −19.65 mV (Figure 9). The high negative zeta potential indicates the non-agglomeration and long-term stability of PFRAE-AgNPs. The zeta potential is a measure of the surface charge of nanoparticles and is used to determine their stability in solution. A high zeta potential value typically indicates that the nanoparticles are highly stable and less likely to aggregate or form clumps. In this study, DLS analysis revealed that the zeta potential value of PFRAE-AgNPs was −19.65 mV, which is a highly negative value and indicates that the nanoparticles are well dispersed and stable in solution. The negative zeta potential value of PFRAE-AgNPs is likely due to the presence of negatively charged functional groups on the surface of the nanoparticles. These groups can repel each other, preventing the nanoparticles from coming into close proximity and aggregating. The stability of PFRAE-AgNPs is particularly important for their potential use in biomedical and pharmaceutical applications, where their stability in solution is critical for their effectiveness [102,103,104,105].

Zeta potential analysis provides insights into the surface charge of nanoparticles, influencing their interaction and dispersion in a liquid medium. A higher magnitude of zeta potential, whether positive or negative, is associated with increased electrostatic repulsion between particles, thereby enhancing stability by preventing aggregation or flocculation.

In our study, we specifically measured the zeta potential to evaluate the stability of the tested nanoparticles. The obtained zeta potential value, which was notably negative in our case (−19.65 mV), indicates a strong repulsive force among particles, suggesting long-term stability of the colloidal system.

It is important to highlight that zeta potential analysis is widely recognized and accepted as a reliable method for assessing the stability of nanoparticles in various applications, including biomedical and environmental contexts. By presenting the zeta potential data, we aimed to provide a comprehensive understanding of the stability profile of the studied nanoparticles.

The long-term stability of PFRAE-AgNPs is important for their potential use in drug delivery systems, where the nanoparticles must remain stable in solution for an extended period of time [104,105]. The high zeta potential value of PFRAE-AgNPs indicates that they are well-suited for such applications, as they are less likely to form aggregates and remain stable in solution over time. In this study, the high negative zeta potential value of PFRAE-AgNPs indicates their non-agglomeration and long-term stability in solution. This is a desirable characteristic for their potential use in biomedical and pharmaceutical applications, particularly in drug delivery systems where stability is critical for their effectiveness [102,103,104,105,106].

### 2.6. Antibacterial Activity of PFRAE-AgNPs

In this study, we assessed the antibacterial activity of streptomycin, PFRAE, 2 mM AgNO_3_, and PFRAE-AgNPs against both Gram-positive bacteria (*S. aureus* and *B. subtilis*) and Gram-negative bacteria (*E. coli* and *P. aeruginosa*). The selection of these microorganisms for antimicrobial activity testing is guided by their pathogenicity, relevance in various environments, representativeness of Gram-positive and Gram-negative bacteria, and potential clinical significance. This comprehensive approach allows us to thoroughly evaluate the antimicrobial potential of the synthesized nanoparticles and contribute to the development of effective antimicrobial agents. The results revealed that all four substances streptomycin, PFRAE, 2 mM AgNO_3_, and PFRAE-AgNPs exhibited effective antibacterial properties against all tested pathogens, albeit with varying degrees of effectiveness against different bacterial species. To gauge this antibacterial activity, we measured the zone of inhibition (ZoI), which quantifies the extent of growth inhibition or retardation in the vicinity of a disc containing the tested substance (Table 1). Streptomycin demonstrated the highest inhibitory effect against all four bacterial strains with the largest inhibition zones. Proving its effectiveness as a broad-spectrum antibiotic, it was potent against all the tested bacteria including *S. aureus* (19.92 ± 1.28 mm), followed by *B. subtilis* (19.13 ± 0.86 mm), *E. coli* (17.86 ± 0.75 mm), and *P. aeruginosa* (17.20 ± 1.05 mm) bacteria. But in recent years, usage became limited due to the rise in antibiotic resistance. PFRAE-AgNPs displayed the second-highest inhibitory effect, particularly effective against *B. subtilis* (15.81 ± 0.68 mm) and *S. aureus* (14.49 ± 1.15 mm) followed by *E. coli* (13.79 ± 0.70 mm) and *P. aeruginosa* (13.06 ± 0.79 mm). Integration of PFRAE with AgNO_3_ enhances antibacterial properties. In comparison, PFRAE alone exhibited a moderate inhibitory effect against the bacterial strains, showing potential for further refinement or combination with other agents for enhanced antibacterial properties. It had a ZoI of 8.56 ± 0.53 mm against *B. subtilis*, followed by a ZoI of 7.57 ± 0.42 mm against *S. aureus*, a ZoI of 7.23 ± 0.65 mm against *E. coli*, and one of 6.83 ± 0.52 mm against *P. aeruginosa*, while 2 mM AgNO_3_ demonstrated a moderate inhibitory effect against the bacterial strains, but slightly higher compared to PFRAE. These silver ions, known for their antibacterial properties, exhibited a ZoI of 10.64 ± 0.72 mm against *S. aureus*, 9.97 ± 0.76 mm against *B. subtilis*, 9.84 ± 1.23 mm against *P. aeruginosa*, and 9.43 ± 0.99 mm against *E. coli*. In summary, streptomycin demonstrated the strongest antibacterial activity, followed by PFRAE-AgNPs, 2 mM AgNO_3_, and PFRAE. Each substance has its strengths and considerations, highlighting the importance of considering factors like potency, broad-spectrum effectiveness, safety, and potential for further refinement in selecting an antibacterial agent for specific applications. Further research and optimization may enhance the effectiveness and safety of these substances. In recent years, the emergence of multidrug-resistant bacteria has spurred the search for innovative and effective antibacterial agents. One promising avenue involves the use of nanotechnology, leveraging the unique properties of nanoparticles. Among these, AgNPs have gained attention for their potent antibacterial properties [107,108]. When combined with plant-based extracts, such as *P. frutescens* extract (PFRAE), AgNPs can exhibit enhanced antibacterial efficacy against a wide range of bacterial strains [109]. PFRAE, derived from *P. frutescens*, possesses inherent antibacterial properties due to its bioactive compounds. When integrated with AgNO_3_, a synergistic effect is observed, resulting in significantly amplified antibacterial activity. The PFRAE-AgNP complex demonstrates substantial inhibitory potential against various bacterial strains. PFRAE-AgNPs show considerable efficacy against *S. aureus*, a Gram-positive bacterium. The unique structural components of Gram-positive bacteria, including a thick peptidoglycan layer, may facilitate higher susceptibility to PFRAE-AgNPs. The nanoparticles likely penetrate the cell membrane more easily due to the inherent permeability characteristics of Gram-positive bacteria [110]. *B. subtilis*, another Gram-positive bacterium, also exhibits notable susceptibility to PFRAE-AgNPs. The nanoparticles may disrupt the cell wall structure of *B. subtilis*, leading to an enhanced inhibitory effect [111]. The interaction could disturb vital cellular processes, hindering bacterial growth and viability. *P. aeruginosa*, a Gram-negative bacterium, is relatively less susceptible to antibacterial agents due to its outer membrane’s impermeability. However, PFRAE-AgNPs still demonstrate a significant inhibitory effect against this strain. The small size and potent antibacterial properties of AgNPs may allow them to breach the outer membrane, reaching the vulnerable intracellular components [112]. *E. coli*, another Gram-negative bacterium, displays moderate susceptibility to PFRAE-AgNPs. While Gram-negative bacteria pose a challenge due to their double-layered membrane, the AgNPs’ ability to induce membrane damage and interfere with essential cellular functions contributes to their inhibitory effect on *E. coli* [113]. In summary, the antibacterial activity of PFRAE-AgNPs showcases a promising avenue in combating bacterial infections. The differential susceptibility observed across bacterial strains underscores the complex interplay between nanoparticle characteristics and bacterial structural variations. Understanding these interactions is vital for optimizing the application of PFRAE-AgNPs in developing effective antibacterial strategies and potentially addressing the global challenge of antibiotic resistance [114]. Further research and tailored approaches are essential to unlock the full potential of PFRAE-AgNPs in the field of antibacterial therapeutics.

AgNPs can cause damage to bacterial cells by interacting with cellular components such as DNA, lipids, and proteins. This damage can lead to bacterial cell death [115]. AgNPs can also disrupt bacterial cell membranes by interacting with the phospholipids and lipopolysaccharides present in the outer membrane of Gram-negative bacteria. This disrupts the integrity of the membrane, leading to leakage of intracellular contents and ultimately cell death [116]. In addition, AgNPs can inhibit bacterial enzymes such as DNA gyrase, which is involved in DNA replication, and ATP synthase, which is involved in energy production. This inhibition can lead to bacterial growth arrest and death [117]. The exact mechanism of action of PFRAE-AgNPs against bacterial pathogens is likely to be complex and multifaceted, involving a combination of the above mechanisms and possibly others [118]. Further research is needed to fully elucidate the mechanism of action of PFRAE-AgNPs and their potential applications as antibacterial agents. The use of natural extracts for the synthesis of antibacterial agents has gained significant attention due to their potential as eco-friendly and sustainable alternatives to synthetic agents. PFRAE, which is derived from the leaves of *P. frutescens*, is known to possess strong antibacterial properties. When combined with AgNPs, the resulting nanocomposite exhibits enhanced antibacterial activity, which can be attributed to the synergistic effects of the two components. Compared to synthetic agents, natural extracts offer several advantages in terms of safety and environmental impact. Natural extracts are generally considered to be safe and non-toxic, with a lower risk of side effects compared to synthetic agents. Additionally, natural extracts are often readily available and can be easily extracted using simple methods, making them a cost-effective alternative to synthetic agents.

We selected both Gram-positive (*B. subtilis* and *S. aureus)* and Gram-negative (*E. coli* and *P. aeruginosa*) bacteria for antimicrobial activity testing based on several key considerations. Firstly, Gram-positive bacteria like *B. subtilis* and *S. aureus* are commonly encountered in various environments and are known to cause a wide range of infections in humans. *B. subtilis*, while generally considered non-pathogenic, can still cause infections, particularly in certain vulnerable populations. *S. aureus*, on the other hand, is a notorious human pathogen responsible for diverse infections and notorious for its ability to develop antibiotic resistance. In contrast, Gram-negative bacteria such as *E. coli* and *P. aeruginosa* are also significant pathogens with unique characteristics. *E. coli* is commonly found in the intestines of humans and animals and can cause severe gastrointestinal illness. *P. aeruginosa*, known for its opportunistic infections, particularly in healthcare settings, poses a serious challenge due to its inherent resistance to many antibiotics and ability to form biofilms. By selecting a combination of Gram-positive and Gram-negative bacteria, we aim to assess the broad-spectrum antimicrobial activity of the synthesized nanoparticles. This approach allows us to evaluate the nanoparticles’ efficacy against different bacterial cell wall structures, as Gram-positive bacteria have a thick peptidoglycan layer, while Gram-negative bacteria have an additional outer membrane. Furthermore, testing against these strains provides insights into the nanoparticles’ potential therapeutic applications and their ability to combat infections caused by clinically relevant pathogens. Additionally, using well-characterized reference strains ensures standardization and facilitates comparison with the existing literature.

Overall, the selection of these microorganisms for antimicrobial activity testing is guided by their pathogenicity, relevance in various environments, representativeness of Gram-positive and Gram-negative bacteria, and potential clinical significance. This comprehensive approach allows us to thoroughly evaluate the antimicrobial potential of the synthesized nanoparticles and contribute to the development of effective antimicrobial agents.

### 2.7. Antioxidant Activity of PFRAE-AgNPs Using DPPH and ABTS Assays

The antioxidant activity of the PFRAE-AgNPs was proved by in vitro radical scavenging assays against both DPPH and ABTS free radicals. The results indicate that PFRAE-AgNPs are effective in scavenging free radicals, and the inhibition of both DPPH and ABTS radicals is dose-dependent. As the concentration of PFRAE-AgNPs increases, the inhibition of free radicals also increases. The maximum inhibition achieved by PFRAE-AgNPs was 92.73% against DPPH radicals and 86.67% against ABTS radicals. The IC_50_ values of PFRAE-AgNPs were found to be 31.6 and 36.2 μg/mL against DPPH and ABTS radicals, respectively. The findings suggest that PFRAE-AgNPs have potent antioxidant activities and could potentially be used in various applications that require antioxidant properties. Further research may be necessary to explore the full range of applications and benefits of PFRAE-AgNPs as antioxidant agents. The mechanism of antioxidant activity of green synthesized AgNPs involves the ability of AgNPs to scavenge free radicals through the transfer of electrons [119]. The surface of AgNPs is abundant in electrons, enabling them to readily donate electrons to free radicals, neutralizing their reactive properties and preventing them from causing damage to cellular components [120]. In the case of PFRAE-AgNPs, the antioxidant activity may be attributed to the presence of bioactive compounds, such as polyphenols (particularly rosmarinic acid), in the plant extract used for their synthesis. These compounds possess antioxidant properties and can bind to the surface of AgNPs, enhancing their scavenging ability and promoting electron transfer. Moreover, the size, shape, and surface properties of nanoparticles can also affect their antioxidant activity [121]. Smaller nanoparticles have a higher surface area-to-volume ratio, which allows for more efficient scavenging of free radicals. The shape of nanoparticles can also influence their antioxidant activity, as nanoparticles with a high aspect ratio, such as nanorods, have been shown to have superior antioxidant properties compared to spherical nanoparticles. In addition to their direct antioxidant activity, AgNPs may also modulate the expression of antioxidant enzymes, such as superoxide dismutase and catalase, which are involved in the cellular defense against oxidative stress. AgNPs can upregulate the expression of these enzymes, leading to a more robust antioxidant response and improved cellular protection against oxidative damage.

The above results demonstrate that PFRAE-AgNPs possess strong antioxidant activity, as evidenced by their ability to scavenge DPPH and ABTS free radicals. This finding is particularly significant because oxidative stress caused by free radicals is implicated in a range of health disorders, including cancer, cardiovascular disease, and neurodegenerative diseases. Therefore, the antioxidant activity of PFRAE-AgNPs suggests that they could have potential therapeutic applications in the prevention and treatment of these diseases [122]. The use of nanoparticles, particularly AgNPs, as antioxidant agents has gained considerable attention in recent years. The high surface area-to-volume ratio of nanoparticles allows for more efficient scavenging of free radicals, while their unique properties make them amenable for use in a wide range of biomedical applications, including drug delivery and diagnostic imaging [123]. In particular, AgNPs have shown promise as effective antioxidant agents due to their ability to generate reactive oxygen species, which can help mitigate oxidative stress. The results of this study also highlight the potential use of plant extracts as natural sources of antioxidants, which can be incorporated into the synthesis of nanoparticles. Plant extracts contain a range of bioactive compounds, such as polyphenols, flavonoids, and tannins, which possess antioxidant properties. Incorporating these compounds into the synthesis of nanoparticles can enhance their antioxidant activity and provide a more natural and sustainable source of antioxidant agents [124].

AgNPs have gained significant attention in biomedical and pharmaceutical research due to their potent antioxidant effects. Their antioxidant applications have resulted in their widespread use in the preparation of food packaging, facial creams, and wound healing ointments. Oxidative stress is implicated in various neurological, cardiovascular, cancer, and aging-related disorders [125]. Antioxidants protect cells from oxidative stress damage, and natural antioxidants are preferred due to their lack of side effects, oral bioavailability, and ease of extraction compared to synthetic compounds [126]. Flavonoids and polyphenolic compounds from different plants and endophytic fungi are known to possess strong natural antioxidant properties, and these compounds can also serve as bioreduction agents for the synthesis and capping of metal nanoparticles. Rosmarinic acid, a natural antioxidant found in essential oils, is commonly used in skin anti-aging formulations and cooking oils to reduce oxidative stress. In this study, rosmarinic acid-capped AgNPs could have significant applications as drug ingredients for the preparation of antioxidant nanoformulations [127]. The capping of AgNPs with rosmarinic acid enhances their antioxidant activity and provides a natural and sustainable source of antioxidant agents. Additionally, the use of AgNPs as drug ingredients in nanoformulations can improve their bioavailability and targeting efficiency, leading to more effective antioxidant therapies. These nanoformulations could potentially be used in the treatment and prevention of oxidative stress-related diseases, such as cancer, cardiovascular disease, and neurodegenerative diseases [128].

### 2.8. Cytotoxicity of PFRAE-AgNPs

**Figure 10:**
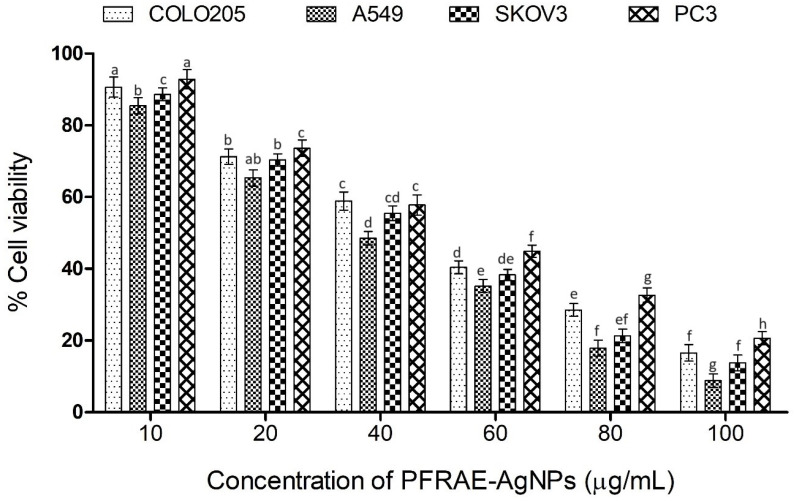
Effect of different concentrations of PFRAE-AgNPs on cell viability across multiple cancer cell lines: statistical analysis using ANOVA with post-hoc testing. Different indicators signify significant differences, while identical indicators indicate comparable viability (significant difference *p* < 0.05).

According to the findings of this study, it can be concluded that PFRAE-AgNPs possess significant cytotoxic activity against various cancer cell lines, including COLO205, A549, SKOV3, and PC3. The maximum inhibition of these cell lines was observed at the highest concentration of PFRAE-AgNPs utilized in this study, with values of 92%, 87%, 84%, and 80% inhibition noted for A549, SKOV3, COLO205, and PC3, respectively (Figure 10). The results also indicated that the viability of cancer cell lines decreased with increasing concentrations of PFRAE-AgNPs, highlighting the strong efficacy of these nanoparticles in inhibiting cancer cell growth and proliferation. Furthermore, this study determined the IC50 concentrations of PFRAE-AgNPs against the aforementioned cancer cell lines, which were found to be 31.25 µg/mL, 43.86 µg/mL, 48.54 µg/mL, and 51.8 µg/mL for A549, SKOV3, COLO205, and PC3, respectively. This implies that these concentrations of PFRAE-AgNPs can effectively inhibit 50% of the cancer cells, indicating their potential for use in cancer therapy and drug delivery systems.

Moreover, the biocompatibility of PFRAE-AgNPs was also evaluated on normal CHO cells, and it was observed that these nanoparticles exhibited negligible cytotoxic effects. This suggests that PFRAE-AgNPs possess favorable compatibility with normal cells, making them a promising option for cancer treatment and drug delivery applications. Overall, these results underscore the potential of PFRAE-AgNPs as potent and biocompatible candidates for cancer therapy and drug delivery, with significant implications for the development of effective and safe cancer treatments.

The level of cytotoxicity of PFRAE-AgNPs against different cell lines can vary due to several factors, including the differences in the cell types, their physiological characteristics, and their sensitivity to nanoparticles [129]. Different cancer cell lines have different properties, such as growth rates, morphologies, and signaling pathways, which can affect their response to nanoparticles. Therefore, the level of cytotoxicity of PFRAE-AgNPs can vary depending on the specific cell line being studied. Moreover, the cellular uptake of nanoparticles can differ depending on the cell type. For example, some cell types may have more efficient uptake mechanisms or more receptors on their surface for nanoparticle binding, leading to increased uptake and subsequent cytotoxicity. Another factor that can affect the cytotoxicity of nanoparticles is the level of oxidative stress in the cell [130]. PFRAE-AgNPs induce the production of reactive oxygen species (ROS) in cancer cells, which can lead to oxidative stress and damage to cellular components such as DNA, proteins, and lipids [131]. The level of oxidative stress in different cell lines can vary, leading to differences in their sensitivity to PFRAE-AgNPs. Furthermore, differences in the size and surface properties of PFRAE-AgNPs can also affect their cytotoxicity. Different cell types may have different sizes and different charges of cell membranes, which can affect the interaction between the nanoparticles and the cell membrane, and therefore the uptake and cytotoxicity of the nanoparticles [132]. There are several reasons why the green synthesized AgNPs may be more effective in cancer treatment: The combination of a natural extract, particularly rosmarinic acid, and AgNPs has been shown to have synergistic effects, which means that the combination is more effective than the individual components alone. Rosmarinic acid is a natural polyphenol with antioxidant and anticancer properties, and AgNPs have been shown to have cytotoxic effects against cancer cells. When combined, the two components work together to enhance their anticancer activity, leading to better outcomes in cancer treatment. One of the potential drawbacks of using physically/chemically synthesized AgNPs in cancer treatment is their potential toxicity to healthy cells [133]. Rosmarinic acid has been shown to have protective effects against oxidative stress and inflammation, which can help to reduce the toxicity of AgNPs. PFRAE-AgNPs are synthesized using a green approach, which means that the nanoparticles are biocompatible and environmentally friendly. In contrast, physically/chemically synthesized AgNPs may require the use of toxic chemicals, which can lead to concerns about their safety and biocompatibility. PFRAE-AgNPs, on the other hand, can be synthesized using natural extracts and environmentally friendly methods, which can reduce their potential toxicity and increase their biocompatibility. PFRAE-AgNPs can be used for targeted delivery of anticancer agents to cancer cells. In summary, the combination of natural extract (rosmarinic acid) and AgNPs in PFRAE-AgNPs has several potential benefits for anticancer activity compared to physically/chemically synthesized AgNPs. These benefits include synergistic effects, reduced toxicity, biocompatibility, and targeted delivery [134]. Therefore, PFRAE-AgNPs may be a promising approach for cancer treatment that warrants further investigation.

**Figure 11:**
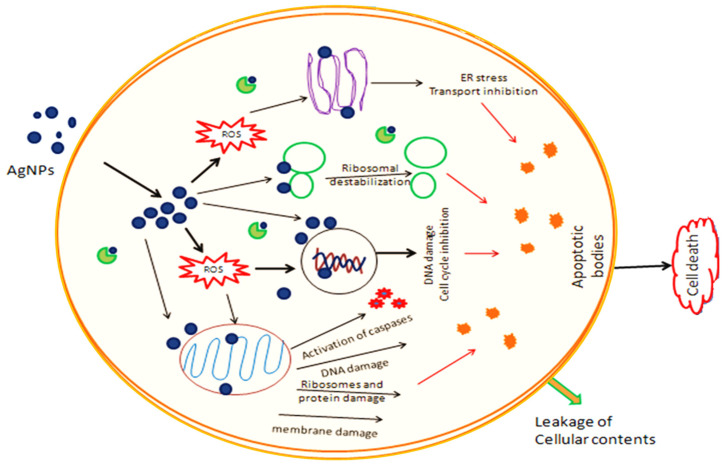
Schematic representation of the mechanism of anticancer activity of silver nanoparticles.

The cytotoxic activity of PFRAE-AgNPs is believed to be due to several mechanisms of action represented in a schematic diagram (Figure 11). 

One mechanism is the induction of reactive oxygen species (ROS) in cancer cells, which leads to oxidative stress and damage to cellular components such as DNA, proteins, and lipids. This, in turn, leads to apoptosis or programmed cell death [131]. Another mechanism of action is the disruption of mitochondrial function in cancer cells, leading to cell death. PFRAE-AgNPs have been shown to inhibit mitochondrial respiration and decrease mitochondrial membrane potential, leading to the activation of apoptotic pathways [135]. Additionally, PFRAE-AgNPs have been shown to induce cell cycle arrest in cancer cells, leading to inhibition of cell proliferation. PFRAE-AgNPs have been found to cause cell cycle arrest at different phases, depending on the cancer cell line being studied [136]. The cytotoxic activity of PFRAE-AgNPs is also believed to be due to their small size and large surface area, which allows them to interact with cellular components more efficiently than larger particles. PFRAE-AgNPs have been shown to penetrate cancer cells and accumulate in the cytoplasm and nucleus, leading to their cytotoxic effects. The findings of this study are promising and suggest that PFRAE-AgNPs have the potential to be developed as novel anticancer agents. Further studies are needed to investigate the mechanisms of action of PFRAE-AgNPs and to evaluate their efficacy and safety in preclinical and clinical studies.

## 3. Materials and Methods

### 3.1. Preparation of PFRAE and Biomimetic Synthesis of PFRAE-AgNPs

The leaves of *P. frutescens* were collected from the cultivating fields near North University of China, Taiyuan, Shanxi Province, China. The leaves were identified by prof. Zhijun Zhang (working on *Perilla frutescens* for the last 30 years). The leaves were deposited in a herbarium (NUCPH1081). The leaves were thoroughly cleaned under running tap water. Finally, the leaves were washed with distilled water. The leaves were dried at room temperature for about a week and then ground into a fine powder. PFRAE was prepared using an ultrasound-assisted extraction method based on a procedure described by Li et al. [69]. Specifically, 10 g of *P. frutescens* leaf powder was mixed with 200 mL of absolute alcohol in a 500 mL conical flask. The flask was placed in an ultrasonicator (Ningbo Scientz SB-5200, Ningbo Scientz Biotechnology, Ningbo, China) and the extraction was carried out at a temperature of 50 °C and a power of 300 W for 45 min. The resulting extract was then purified using silica gel column chromatography, and HPLC analysis was performed to confirm the presence of rosmarinic acid. The purified extract was named PFRAE and was subsequently used for the synthesis of PFRAE-AgNPs. To synthesize PFRAE-AgNPs, 10 mL of PFRAE (1 mL/g DW of perilla powder which constitutes 0.798 mg of RA/g DW of perilla powder) was added to 90 mL of AgNO_3_ (2 mM) and the mixture was boiled at 50 °C for 1 h and then allowed to cool in a dark chamber for 30 min. It was observed for a color change from light yellow to dark brown.

### 3.2. Spectral Characterization of PFRAE-AgNPs

UV–Vis analysis is a spectroscopic technique used to measure the absorption and transmission of light in the visible and ultraviolet region of the electromagnetic spectrum. The analysis was performed on a dark brown solution of PFRAE-AgNPs using a PerkinElmer Ltd., Instrument (Waltham, MA, USA) with a wavelength range of 200–800 nm. The colloidal solution of PFRAE-AgNPs was then separated using centrifugation (15,000 rpm for 15 min), and the resulting pellet was dried to obtain a fine powder for further spectral characterization. Fourier transform infrared spectroscopy (FTIR) is a technique used to identify the functional groups present in a sample by measuring the absorption of infrared radiation from the sample. Functional group analysis of PFRAE-AgNPs was carried out using FTIR (Alpha interferometer, Bruker, Karlsruhe, Germany). X-ray diffraction (XRD) is a technique used to determine the crystal structure and phase of a sample by measuring the diffraction of X-rays by the sample. In this case, the analysis was performed using a Bruker D8 advance instrument, (Karlsruhe, Germany). Transmission electron microscopy (TEM) is a technique used to visualize the size and shape of nanoparticles by transmitting a beam of electrons through the sample. In this study, TEM analysis (JEM-2100 Plus Electron Microscope, JEOL Ltd., Tokyo, Japan) was carried out to reveal the sizes and shapes of PFRAE-AgNPs. Dynamic light scattering (DLS) is a technique used to measure the size distribution and zeta potential of nanoparticles in a colloidal solution. In the present study, DLS analysis was carried out using a Brookhaven instrument (Brookhaven Instruments, Holtsville, NY, USA) to determine the zeta potential values and dispersity index of PFRAE-AgNPs.

### 3.3. Antibacterial Activity of PFRAE-AgNPs

The antibacterial activity of PFRAE-AgNPs was evaluated using the disc diffusion assay against both Gram-positive bacterial (*Bacillus subtilis* and *Staphylococcus aureus*) and Gram-negative bacterial (*Pseudomonas aeruginosa* and *Escherichia coli*) pathogens [66]. In this assay, 150 µL of each bacterial culture was evenly spread on nutrient agar plates. Subsequently, sterile paper discs were saturated with various test samples of 25 µL, including streptomycin, PFRAE, 2 mM AgNO_3_, and PFRAE-AgNPs. These discs were then placed onto the nutrient agar plates. The plates were incubated at 37 °C for 24 h, allowing bacterial growth. After incubation, the inhibition zones were observed and measured.

### 3.4. Antioxidant Activity of PFRAE-AgNPs

The DPPH and ABTS radical scavenging assays were employed to assess the antioxidant activity of PFRAE-AgNPs.

#### 3.4.1. DPPH Scavenging Assay

To prepare the 1 mM DPPH stock solution, 4 mg of DPPH was dissolved in 100 mL of methanol. Various concentrations of the test samples (10, 20, 40, 60, 80, and 100 µg/mL) were separately prepared in methanol, each with a final volume of 1 mL. Subsequently, 1 mL of the methanolic solution containing the test sample was mixed with 2 mL of the DPPH solution. This mixture was then incubated in the dark at room temperature for 1 h [69]. Following the incubation period, the absorbance values were measured at 517 nm, and the DPPH scavenging activity was calculated using the following formula: % DPPH scavenging = [(Absorbance of Control − Absorbance of Test)/Absorbance of Control] × 100.

#### 3.4.2. ABTS Scavenging Assay

To prepare the ABTS working solution, a mixture containing 7 mM of ABTS and 2.45 mM of potassium persulfate was combined in a conical flask and left to incubate for 16 h. Following this incubation period, the resulting solution was diluted with 70% ethanol until it reached an absorbance of 0.02 when measured at 734 nm. Subsequently, for the antioxidant activity assay, 0.1 mL of various test sample concentrations (10, 20, 40, 60, 80, and 100 µg/mL) was added to 0.9 mL of the ABTS working solution. This mixture was thoroughly vortexed and then incubated in darkness for 30 min [69]. After the incubation period, absorbance values were measured at 734 nm, and the percentage of ABTS scavenging activity was calculated using the following formula: % ABTS scavenging activity = [(Absorbance of Control − Absorbance of Test)/Absorbance of Control] × 100.

### 3.5. Cytotoxicity of PFRAE-AgNPs

The cytotoxicity of PFRAE-AgNPs was evaluated using the 3-(4,5-dimethylthiazo-2-yl)-2,5-diphenyltetrazolium bromide (MTT) assay [137]. PFRAE-AgNPs were tested for their cytotoxic effects on various cancer cell lines, including COLO205, PC-3, A549, SKOV3, as well as a normal cell line (CHO). In this assay, actively growing cells (5 × 10^−3^/100 µL) were initially seeded and allowed to incubate overnight at 37 °C in a CO_2_ (5%) chamber. After the overnight incubation, the cells were exposed to different concentrations (5, 10, 20, 40, 60, 80, and 100 µg/mL) of PFRAE-AgNPs. Following a 24 h treatment period, 10 μL of MTT solution (5 mg/mL) in phosphate-buffered saline (pH 7.4) was added to each well and allowed to incubate for an additional 3 h. After this incubation, the culture medium containing MTT was removed, and the formazan blue crystals that had formed were dissolved in dimethyl sulfoxide (DMSO). Subsequently, the absorbance values were measured at 570 nm. Using these measurements, the cell viability percentage was calculated, and the IC_50_ values, which represent the concentration at which 50% of the cells were affected, were determined.

## 4. Conclusions

In conclusion, the synthesis of silver nanoparticles using a rosmarinic acid extract from *Perilla frutescens* has shown to be an effective and eco-friendly method. The characterized PFRAE-AgNPs exhibited unique properties, including their small size, spherical morphology, crystalline structure, high stability, and negative zeta potential, making them promising candidates for diverse biomedical applications. The antibacterial activity of PFRAE-AgNPs suggests their potential in treating bacterial infections, while their antioxidant properties highlight their possible use in treating oxidative stress-related diseases. Furthermore, their demonstrated anticancer activity against a range of cell lines makes them a promising candidate for cancer therapy. The small size and high stability of PFRAE-AgNPs could enable them to be used in drug delivery systems, enhancing drug efficacy, and reducing side effects. The biocompatibility of PFRAE-AgNPs towards normal cells is an important factor for their potential use in biomedical applications. It indicates that these nanoparticles have low toxicity and minimal harmful effects on healthy cells, which is a crucial aspect for developing safe and effective drug delivery systems. The use of biocompatible AgNPs in cancer therapy can help reduce the side effects associated with conventional chemotherapy, which often damages both cancerous and healthy cells. Therefore, the results of this study provide valuable insights into the potential use of PFRAE-AgNPs as a safe and effective tool for cancer therapy and drug delivery systems. Additionally, their cost-effective synthesis method suggests that they can be easily scaled up for industrial production. In summary, this study provides new insights into the potential of using natural plant extracts for the synthesis of nanoparticles with diverse biomedical applications. The unique properties of PFRAE-AgNPs make them promising candidates for the development of novel therapeutic agents and drug delivery systems.

## Figures and Tables

**Table 1:** Antibacterial activity of tested substances against different bacterial strains. This table presents the zone of inhibition (ZoI) in millimeters (mm) for streptomycin, PFRAE, 2 mM AgNO_3_, and PFRAE-AgNPs against *S. aureus*, *B. subtilis*, *P. aeruginosa*, and *E. coli*.

Tested Substance	*S. aureus* ZoI (mm)	*B. subtilis* ZoI (mm)	*P. aeruginosa* ZoI (mm)	*E. coli* ZoI (mm)
Streptomycin	19.92 ± 1.28	19.13 ± 0.86	17.20 ± 1.05	17.86 ± 0.75
PFRAE	7.57 ± 0.42	8.56 ± 0.53	6.83 ± 0.52	7.23 ± 0.65
2 mM AgNO_3_	10.64 ± 0.72	9.84 ± 1.23	9.97 ± 0.76	9.43 ± 0.99
PFRAE-AgNPs	14.49 ± 1.15	15.81 ± 0.68	13.06 ± 0.79	13.79 ± 0.70

## Data Availability

Data are contained within the article.
